# The Mediating Role of Self-Concept and Coping Strategies on the Relationship Between Attachment Styles and Perceived Stress

**DOI:** 10.5964/ejop.v14i4.1508

**Published:** 2018-11-30

**Authors:** Rıza Bayrak, Murat Güler, Nesrin Hisli Şahin

**Affiliations:** a Department of Business Administration, National Defense University, Ankara, Turkey; b Department of Business Administration, Niğde Ömer Halisdemir University, Niğde, Turkey; c Department of Psychology, Başkent University, Ankara, Turkey; Department of Psychology, Webster University Geneva, Geneva, Switzerland; Università di Roma LUMSA, Rome, Italy

**Keywords:** attachment, self-concept, coping, stress factors, stress symptoms

## Abstract

The aim of this study was to examine the role of attachment style, self-concept, and coping strategies, in order to explain the differences in perceived stress factors and stress symptoms, in a mediation model. Participants were 515 university students (302 female and 213 male) aged 17-28 years. The assessment instruments were: Social Comparison Scale, Ways of Coping Scale, Experiences in Close Relationships Scale-II, Brief Symptom Inventory and University Students Stress Factors Scale. The results indicated that the effect of anxious attachment on perceived stress factors and stress symptoms was partially mediated by self-concept and coping styles. Our findings revealed that the level of anxious attachment is an important factor to explain perceived stress and stress-related variables.

Stress, as a relationship between the individual and an environmental change, appraised as endangering well-being, and taxing or exceeding the individual’s coping resources (Lazarus & Folkman, 1986), affects individuals differently, due to their differences in their assessment of the situation and coping strategies employed (Folkman, Lazarus, Gruen, & Delongis, 1986; Lazarus, 1993). The assumptions of the individual regarding his or her own coping skills play an important role in the evaluation and interpretation of this relationship between the individual and the environment (Park & Folkman, 1997). Accordingly, in order to understand the link between the stress sources and the stress response of the individual, it is important to take into consideration, their personality traits (Lazarus, 1993), such as attachment styles and self-concept.

## The Theoretical Background

There is some evidence regarding the significance of the attachment styles as a personality trait, to explain the concept of stress. Bowlby (1973) notes that, secure attachment relationship between the caregiver and the child allows the child, to develop a healthy psychological development, and that these early relational experiences shape the interpersonal processes of the individual's later life. If the basic requirements for safe attachment during infancy and childhood are not met adequately, psychopathological problems may develop. In previous studies, attachment styles have been associated with various psychopathologies such as personality disorders (Aaronson, Bender, Skodol, & Gunderson, 2006; Jellema, 2000; Mauricio, Tein, & Lopez, 2007), depression (Carnelley, Pietromonacó, & Jaffe, 1994; Wei, Mallinckrodt, Larson, & Zakalik, 2005) and anxiety disorders (Doron & Kyrios, 2005).

Attachment behavior, as defined by Bowlby (1973), is the tendency of the human being as a mammal to get close to an adult that he or she accepts as a shelter/protection. The quality of this attachment relationship has an important role in personality development and affects the individual’s whole life. Attachment styles, starting to develop between the baby and the caretaker just after the birth, can be conceptualized like a prototype or a schema for interpersonal relations in the future (Ainsworth et al., 1978; Morris, 1982). Sperling and Berman (1994) define adult attachment, as a tendency and an effort to establish closeness with other people, they feel safe and secure with. Due to this tendency, which is also termed as secure-insecure attachment styles observed in all people, mental schemas or mental representations (inner working models) about the world and other people develop (Sperling & Berman, 1994). These mental schemas determine how people perceive the interpersonal events they experience. In early studies, besides secure attachment style, anxious-resistant and avoidant attachment styles which depend on Ainsworth et al.’s work (1978) were in focus of research. Bartholomew and Horowitz (1991) suggested a four-category model based on the perception of self and others dimensions. According to that model, by dichotomizing these dimensions as positive or negative four attachment styles were defined as secure, preoccupied, dismissive avoidant and fearful avoidant. Practically this model divided avoidant attachment into two parts. Brennan, Clark, and Shaver (1998) made a large-scale factor analytic study on existing self-measures of adult attachment and suggested two types of attachment, avoidance (discomfort with closeness and depending to others) and anxiety (fear of rejection and abandonment).

Previous studies on attachment styles have shown that each attachment style is associated with different personality traits, and different interpersonal problems (Sümer & Güngör, 1999). When the relationship between attachment styles and stress is examined, it is seen that securely attached individuals show less anxiety and depression symptoms, compared to insecurely attached ones, and they perceive more social support in their environment and get more satisfaction (Priel & Shamai, 1995). Secure attachment is said to be negatively related to perceived stress and psychological symptoms; whereas insecure attachment is positively related to both (McCarthy, Lambert, & Moller, 2006; Pielage, Gerlsma, & Schaap, 2000). It is asserted that securely attached individuals are high in self-confidence and low in alienation compared to others, while insecurely attached individuals show higher anxiety and depression symptoms (Fortuna & Roisman, 2008; Muris, Meesters, Melick, & Zwambag, 2001). However, when the insecure attachment is investigated according to its sub-divisions, some studies indicate that only anxious attachment is found to be positively related to perceived stress, while no relationship was found between avoidant attachment and perceived stress (Maunder, Lancee, Nolan, Hunter, & Tannenbaum, 2006). This finding was interpreted as a result of the suppressing tendency of the avoidantly attached individuals (Hunter et al., 2016; Teeruthroy & Bhowon, 2012). Anxious attachment was also found to be positively related anxiety symptoms and predict depressive symptoms (Hankin, Kassel, & Abela, 2005). It was stated that those with avoidant attachment styles with high anxiety, revealed more psychological symptoms compared to the ones with lower anxiety; while those with both anxious and avoidant attachment styles experienced more intense anxiety, depression and post-traumatic stress symptoms (Elwood & Williams, 2007; Neria et al., 2001; Renaud, 2008).

In some studies, examining the relationship between attachment and stress, it is seen that coping strategies are used as a mediating variable (Lopez, Mauricio, Gormley, Simko, & Berger, 2001; Merlo & Lakey, 2007; Wei, Heppner, & Mallinckrodt, 2003). It is stated that there is a significant positive relationship between anxious and avoidant attachment styles and ineffective coping; additionally, ineffective coping predicts stress symptoms (Lopez et al., 2001). Wei et al. (2003) suggest that while coping strategies completely mediate the relationship between anxious attachment and stress; coping has a partial mediating role in the relationship between avoidant attachment style and stress. In some studies, it was reported that securely attached individuals prefer active coping strategies against stress (effective coping); whereas insecurely attached ones prefer to use avoidant coping strategies (Li, 2008; Seiffge-Krenke & Beyers, 2005). In another study, it was stated that those who have dismissive avoidant attachment style, employ less social and emotional support as a coping strategy (Hawkins, Howard, & Oyebode, 2007). Moreover, it was suggested that there is a positive relationship between secure attachment and seeking social support and problem-focused coping. Additionally, this positive relationship also appears between anxious attachment and avoidant coping style. Anxiously attached individuals perceive social encounters as more stressful than the others and tend to avoid from them (Schottenbauer et al., 2006).

According to some studies on the relationship between self-concept, attachment, and stress, securely attached university students were found to have a more positive self-image compared to insecurely attached ones (Damarlı, 2006); and with increasing stress, while positive self-image decreases, the use of ineffective coping mechanisms increases (Tuğrul, 1994). It is also suggested that self-esteem is a significant predictor of coping strategies (Avşaroğlu & Üre, 2007); moreover, as stress factors increase, positive self-concept decreases, while vulnerability to stress and stress symptoms increase (Kulaç, 2004). In some studies examining the relationship between perceived stress factors, stress symptoms and coping, it was seen that the individuals using more effective coping mechanisms, experienced lower stress symptoms (Austin, Shah, & Muncer, 2005; Lee & Larson, 1996; Şahin, Güler, & Basım, 2009; Tully, 2004); and that there was a negative correlation between effective coping methods and stress factors (Basut & Erden, 2005; Sığrı, 2007), and a significant positive correlation between stress factors and stress symptoms (Greene, Walker, Hickson, & Thompson, 1985; Şahin et al., 2005).

### The Current Study

**Figure 1:**
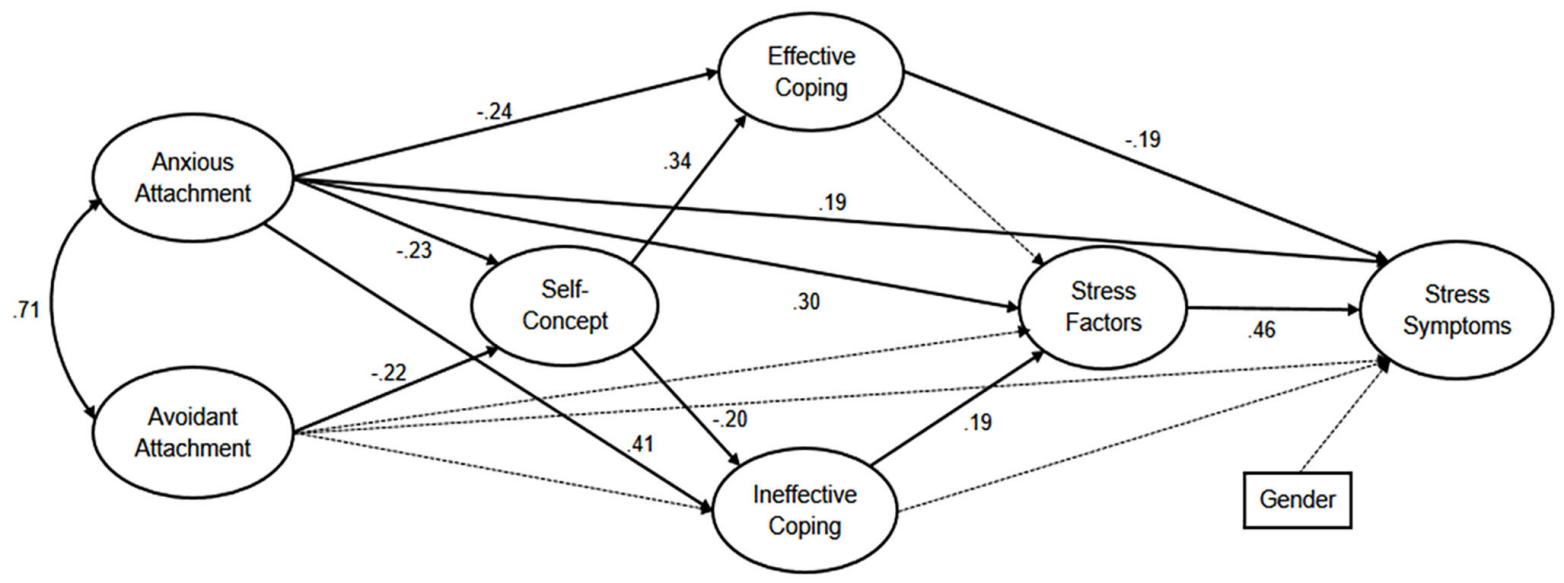
Path analysis model and analysis results.

In this context, assuming that stress symptoms are a function of the interaction between personality traits and perceived stress factors (Şahin, Basım, & Akkoyun, 2011); and that different attachment types may be related to different personality traits (Sümer & Güngör, 1999) and experienced stress symptoms (Pielage et al., 2000; Priel & Shamai, 1995); the present study was designed to investigate the mediatory role of attachment types, self-concept and coping strategies on the perceived stress factors and stress symptoms, as depicted in Figure-1. As mentioned above, even though these relationships were analyzed separately in the previous studies, as far as we know, they are analyzed as a group of variables in a model, for the first time in the current study.

## Method

### Participants

University students (575) studying in four different public and private universities in Ankara, participated in the study, giving an informed consent. This was a sample of convenience. Totally, 60 forms were excluded from the analysis due to missing items in the scales or unfilled scales in the testing battery. As a result, 515 participants were analyzed. The age range was between 17 and 28 (*M* = 21, *SD* = 2). In terms of gender, 213 participants were male (41.4%) and 302 were female (58.6%).

### Procedure

An assessment battery was formed by gathering the five scales to measure the research variables, self-concept, stress (factors, symptoms), coping, and attachment. In order to reduce the effects of possible factors out of the scope of the current study, the university students were considered as a convenient sample, since they are in a similar environment, and relatively more homogeneous community, compared to the society in general. Participants were a convenience sample from university students. The survey was administered to 10-30-person groups in classroom conditions and lasted 30 minutes on average. Statistical analyses of the means, standard deviations, correlations among the research variables and their mediating roles, were conducted by IBM SPSS 23, and the path analysis was conducted by using IBM AMOS 23.

### Measures

#### Experiences in Close Relations Inventory-II (ECRI-II)

This is a revision based on the item-response theory (Fraley & Shaver, 2000), of the original 18-item Experiences in Close Relations Inventory, originally developed by Brennan, Clark, and Shaver (1998), to measure individual’s attachment on two subscales, namely, “anxious” and “avoidant” attachment styles (Hambleton, Swaminathan, & Rogers, 1991). The anxious attachment dimension (each item is evaluated on a 7-point Likert type scale) indicates to an attachment anxiety, as originating from oversensitivity to rejection and feelings of abandonment, experienced in close relationships; on the other hand, avoidant anxiety dimension, indicates to a discomfort felt in being close to, or dependent on others (Sümer, 2006). Turkish adaptation of the Experiences in Close Relation Inventory-II was conducted by Selçuk, Günaydin, Sümer, and Uysal (2005). The Cronbach’s Alpha coefficients of anxious and avoidant attachment styles were reported as α = .86 and .90 respectively (Selçuk et al., 2005). In the current study, a confirmatory factor analysis (CFA) was conducted and the results confirmed the two-dimensional structure of the Scale. The Goodness of fit values was found to be acceptable (χ^2^/*SD* = 2.274, RMSEA= .05, CFI = .92, GFI = .91), after the exclusion of the 9th, 19th, 29th, 14th and 28th items from the scale. These five items were found to be related other constructs in CFA and needed to be excluded from measurement. Three of the items also had the lowest factor scores in the original adaptation study (Selçuk et al., 2005). The internal consistency coefficients of the anxious and avoidant attachment dimensions were found as α = .85 and α = .88 respectively.

#### Social Comparison Scale

It is a self-assessment scale, designed to determine the perceptions of an individual when comparing of himself or herself to others, on various dimensions. The scale was originally developed by Gilbert, Allan, and Trent (1991) as 5-item form, and later, was adapted into Turkish with the addition of some items by Şahin and Şahin (1992), turning it into an 18-item scale. It consists of 18 bipolar items, evaluated on a 6-point Likert-type scale. An example item is “incompetent” or “competent”. High scores indicate positive self-perception, whereas low scores indicate negative self-perception (Savaşır & Şahin, 1997). The internal consistency of the Turkish adapted form was found as α = .89 (Şahin, Durak, & Şahin, 1993). In the current study, the internal consistency coefficient was found to be α = .86. The CFA analysis result confirmed the single dimensional structure of the scale, and the Goodness of fit indexes was acceptable (χ^2^/*SD* = 1.876, RMSEA = .04, CFI = .98, GFI = .96).

#### University Students’ Stress Factors Scale

This is a 160-item scale, developed specifically for the Turkish culture by Şahin et al. (2005), to determine the factors related to stress among university students. The eight subscales of the Scale are self-related problems, responsibilities, social problems, academic problems, problems related to behaviors contrary to traditional values, economic problems, problems about close relationships and problems about being away from relatives (family, friends) (Şahin et al., 2005). The participants were asked to rate how the given statements effected them in the last three months. An example item is “Living away from family”. The internal consistency coefficients for the sub-dimensions were found to be between α = .81 and α = .93, and for the total scale, it was α = .97, in the current study. Since the ratio of the sample size to the number of items was not enough to perform a CFA, criterion-dependent validity was considered as the scale validity criterion. These were found to be as *r* = .59 (*p* < .001) (with stress symptoms); *r* = .40 (*p* < .001) (with anxious attachment); and *r* = .34 (*p* < .001) (with ineffective coping) (Büyüköztürk, 2010).

#### Coping With Stress Scale

The 66-item original scale, developed by Lazarus and Folkman (1984), was adapted for the Turkish university students, by Şahin and Durak (1995), and transformed into a 30-item short form. The scale is based on the assumption that individuals have unchanging coping strategies in different situations. The coping methods were separated into two-factor sub-scales as, “problem-oriented / active / effective coping” and “emotion-oriented / passive / ineffective coping”. An example item is “When I have a problem I try to be optimist”. The reliability coefficients reported for the problem-oriented/active methods was α = .82 and for emotion-oriented/passive methods α = .78 (Şahin et al., 2009). In the current study, the results of the CFA confirmed the two-dimensional structure of the scale as effective and ineffective coping. The goodness of fit index values (χ^2^/*SD* = 2.152, RMSEA = .05, CFI = .90, GFI = .91) were found to be acceptable. The internal consistency coefficients for effective coping and ineffective coping were α = .78 and α =.75, respectively.

#### Brief Symptom Inventory

This is 53-item, a short form of the SCL-90-R inventory developed by Derogatis (1992), for the purpose of screening various psychological symptoms. A new factor analysis was conducted on a Turkish sample by Şahin and Durak (1994) and five factors were found. These factors are: Anxiety, Depression, Negative Self, Somatization, and Hostility. The total score received from the four-point Likert type scale indicates to the intensity of the individual's stress symptoms. An example item is “Feeling strained and uneasy”. In the current study, the internal consistency coefficients of the sub-dimensions were found to be between α = .73 and α =.85, and as α = .95 for total scale; the Goodness of fit values for the one-dimensional structure of the scale (χ^2^/*SD* = 1.596, RMSEA = .03, CFI = .95, GFI = .90) were found to be as acceptable.

## Results

A Pearson correlation analysis was performed to see the relationships among the research variables. Table 1 shows the descriptive statistics and the correlations. Except for gender, all the variables correlated with each other significantly (*p* < .001). The highest correlation was found between anxious attachment and avoidant attachment (*r* = .71) Anxious attachment was also moderately positively correlated with ineffective coping (*r* = .49) and stress symptoms (*r* = .44). Also, stress symptoms was positively correlated with perceived stress factors (*r* = .59), ineffective coping (*r* = .40) and negatively correlated with effective coping (*r* = -.39). Gender (being female) slightly negatively related with avoidant attachment (*r* = -.12, *p* < .01) and effective coping (*r* = -.11, *p* < .01).

**Table 1:** Descriptive Statistics and Correlations

Variables	*M*	*SD*	1	2	3	4	5	6	7
1. Gender (M=1, F=2)	-	-	-						
2. Anxious Attachment	3.35	1.20	-.06	-					
3. Avoidant Attachment	2.54	0.88	-.12*	.71**	-				
4. Self-Concept	0.95	0.13	.01	-.39**	-.38**	-			
5. Effective Coping	1.74	0.47	-.11*	-.37**	-.27**	.43**	-		
6. Ineffective Coping	0.53	0.46	.08	.49**	.28**	-.36**	-.70**	-	
7. Stress Factors	1.40	0.52	.04	.40**	.25**	-.15**	-.28**	.34**	-
8. Stress Symptoms	0.85	0.52	.03	.44**	.31**	-.25**	-.39**	.40**	.59**

*Note.* *n* = 515.

**p < .01.

In order to examine the direct and indirect effects of the research variables within the research model, a path analysis, which enables to define a variable as both dependent and independent variable in the same analysis, was conducted. The path analysis working on the principles of multiple regression logic is defined as a model in which direct and indirect statistical effects can be tested. In the structural equation modeling studies with latent variables, the sample size needs to be at least 5 to 10 times the number of observed variables according to the distribution features of the data (Gürbüz & Şahin, 2016). Since the total number of items (total 297 items) of the scales used in the present study is considerably large, in terms of the required sample size for path analysis, the factor scores were used as the observed variables and the path analysis was performed with these variables. In a path analysis which is performed this way, despite the limitations of the measurement errors, with respect to the aim of the research, direct and indirect effects can be analyzed all together on a single path analysis, instead of separately conducted much more multiple regression analyses to examine them. Furthermore, to figure out the latent variables scores, instead of a simple average of the total item scores, regression imputation method in AMOS (Arbuckle, 2014), which takes into account the effect values of each item on the scale as identified in the confirmatory factor analysis, was used.

Anxious attachment and avoidant attachment were included as exogenous variables; self-concept, coping strategies, stress factors, and stress symptoms were included as endogenous variables in the model. In the final model, in which nonsignificant (*p* > .05) paths were not included, the goodness of fit values (χ^2^/*SD* = 2.275, RMSEA = .05, CFI = .99, GFI = .99) were found to be at a “good fit” level, which means the model fits the data well (Gürbüz & Şahin, 2016; Meydan & Şeşen, 2011).

The path analysis model and analysis results for significant (*p* < .05) parameter estimates of the direct and indirect effects are shown in Figure 1. Except for the absence of a significant relationship between effective coping and perceived stress factors, expected significant effects were observed at each step in the model. The results indicated that, anxious attachment (β = -0.23, *p* < .001) and avoidant attachment (β = -0.22, *p* < .001) were negatively related to self-concept at a significant level. Self-concept seemed to be significantly and positively related to effective coping (β = 0.34, *p* < .001), but negatively related to ineffective coping (β = -0.20, *p* < .001). The results also suggested that ineffective coping was positively related to perceived stress factors, while effective coping was negatively related to stress symptoms (β = -0.19, *p* < .05). Perceived stress factors were positively related to stress symptoms (β = 0.46, *p* < .001). It was also observed that anxious attachment was significantly and positively related to ineffective (β = 0.41, *p* < .001), negatively related to effective coping (β = -0.24, p < .01); while it was positively related to perceived stress factors (β = 0.30, *p* < .001) and stress symptoms (β = 0.19, *p* < .001). Gender entered as a control variable into the analysis and no significant (*p* < .05) effect on stress symptoms was found.

Further analyses were performed in the following order, to clarify whether the explored significant interactions included a mediating role or not. In a simple mediation analysis, frequently the causal step approach (Baron & Kenny, 1986) is used and the product of coefficients approach (Sobel, 1982) is employed, to test the validity of the conclusions reached (Burmaoğlu, Polat, & Meydan, 2013; Hayes, 2009). However, it is stated that both of these approaches should not be conducted due to the problems in their basic assumptions. Instead of these methods, use of bootstrap confidence interval method is suggested, which allows examining the effects of more than one mediating variable at the same time, despite lower sample sizes (Preacher & Hayes, 2008; Shrout & Bolger, 2002).

Nevertheless, since a specific mediating role actually cannot be individually assessed (Preacher & Hayes, 2008, p. 887), and since we needed to examine not only the mediating role of each variable (self-concept, coping strategies and perceived stress factors) separately, and also controlling for the mediating role of other mediators, the bootstrap bias-corrected confidence interval (BC 95% CI) method (Preacher & Hayes, 2008; Shrout & Bolger, 2002) (which enables to examine more than one mediator variable simultaneously) was used. The size of the bootstrap resample was determined as 5000 (Preacher & Hayes, 2008). The results are presented in Table 2.

**Table 2:** Mediation Analyses Results

Effect	Point Estimate	*SD*	BC %95 CI	
			Lower	Upper
Effective Coping ← Anxious Attachment				
Total Effect (c)	-.123	.018	**-.157**	**-.088**
Direct Effect(c**’**)	-.093	.017	**-.126**	**-.060**
Indirect Effect (axb)	-.031	.009	**-.051**	**-.015**
Ineffective Coping ← Anxious Attachment				
Total Effect(c)	.174	.016	.141	.204
Direct Effect(c**’**)	.155	.017	.123	.188
Indirect Effect(axb)	.018	.006	.008	.033
Effective Coping ← Avoidant Attachment				
Total Effect(c)	-.038	.012	**-.065**	**-.019**
Direct Effect(c**’**)	.000	.000	.000	.000
Indirect Effect(axb)	-.038	.012	**-.065**	**-.019**
Ineffective Coping ← Avoidant Attachment				
Total Effect(c)	.023	.008	**.010**	**.041**
Direct Effect(c**’**)	.000	.000	.000	.000
Indirect Effect(axb)	.023	.008	**.010**	**.041**
Perceived Stress Factors ←Anxious Attachment				
Total Effect(c)	.169	.017	**.135**	**.202**
Direct Effect(c**’**)	.132	.019	**.095**	**.168**
Total Indirect Effect(Σ a_i_xb_i_xd_i_)	.038	.010	**.019**	**.059**
Perceived Stress Factors ←Avoidant Attachment				
Total Effect(c)	.005	.002	**.002**	**.010**
Direct Effect(c**’**)	.000	.000	.000	.000
Total Indirect Effect(Σ a_i_xb_i_xd_i_)	.005	.002	**.002**	**.010**
Perceived Stress Factors ← Self-Concept				
Total Effect(c)	-.151	.049	**-.265**	**-.068**
Direct Effect(c**’**)	.000	.000	.000	.000
Indirect Effect(axb)	-.151	.049	**-.265**	**-.068**
Stress Symptoms ← Anxious Attachment				
Total Effect(c)	.186	.017	**.152**	**.218**
Direct Effect(c**’**)	.081	.016	**.049**	**.110**
Total Indirect Effect(Σ a_i_xb_i_xd_i_)	.105	.012	**.084**	**.129**
Stress Symptoms ← Avoidant Attachment				
Total Effect(c)	.010	.004	**.005**	**.020**
Direct Effect(c**’**)	.000	.000	.000	.000
Total Indirect Effect(Σ a_i_xb_i_xd_i_)	.010	.004	**.005**	**.020**
Stress Symptoms ← Self-Concept				
Total Effect(c)	-.319	.068	**-.468**	**-.202**
Direct Effect(c**’**)	.000	.000	.000	.000
Total Indirect Effect(Σ a_i_xb_i_xd_i_)	-.319	.068	**-.468**	**-.202**
Stress Symptoms ← Ineffective Coping				
Total Effect(c)	.101	.026	**.052**	**.154**
Direct Effect (c**’**)	.000	.000	.000	.000
Indirect Effect(axb)	.101	.026	**.052**	**.154**

*Note*. *n* = 515 (5.000 Bootstrap sample), BC = Bias corrected, a = effect of X on M, b and d = effect of M on Y, c = total effect of X on Y, c’= direct effect of X on Y.

Below the bootstrap corrected confidence interval (%95) column (Table 2), there are two columns that show the lower and upper values with the effect sizes, within 95% confidence interval. If the values between the two limits do not contain "0", then the examined indirect effect is considered to be significantly different from zero (Preacher & Hayes, 2008; Shrout & Bolger, 2002). Our analyses showed that all the indirect effects examined in the model were significant in terms of the 95% confidence interval. Significant effects were marked as bold on the table. It was also seen that most of (B = 0.105) the total effect (B = 0.186) of anxious attachment on stress symptoms, occurred indirectly, through self-concept, coping, and perceived stress factors, whereas some of it took place directly (B = 0.081). According to this result, it can be said that self-concept, coping and perceived stress factors may have a partial mediator role in the relationship between anxious attachment and stress symptoms. It was also found that self-concept, coping and perceived stress factors fully mediated the relationship between avoidant attachment and stress symptoms (B = 0.010). While coping and perceived stress factors fully mediated the “self-concept and stress symptoms relationship” (B = -0.319), the “ineffective coping and stress symptoms relationship” (B = 0.101), was found to be fully mediated by perceived stress factors.

## Discussion

In this study, a mediation model was used to examine the effects of the study variables, i.e., attachment styles, self-concept, coping strategies and perceived stress factors, to predict stress symptoms.

The main result of the study is that, perceived stress factors and stress symptoms significantly differ according to personality characteristics and that, the anxious attachment has a greater predictive power on stress symptoms when its direct and indirect statistical effects are taken into consideration. In other words, it can be assumed that those who have high levels of anxious attachment may experience greater levels of stress compared to others. It can also be suggested that, besides the direct effect of anxious attachment on stress symptoms, it has an indirect effect through self-concept, coping methods and perceived stress factors. In considering the relationship of anxious attachment with other variables, it can be assumed that anxiously attached individuals have a more negative self-concept; they use more ineffective coping strategies compared to the effective ones; they perceive more stress factors, and also as a result, they experience a greater intensity of stress symptoms. However, it is noteworthy that, although avoidance type attachment does not have a direct significant effect on stress symptoms, it has an indirect significant effect on stress symptoms through self-concept. Similar with the current study’s findings, in the related literature, it is stated that there is a significant and positive correlation between anxious attachment and perceived stress; however, avoidant attachment does not seem to be significantly related to stress symptoms (Elwood & Williams, 2007; Fortuna & Roisman, 2008; Hankin et al., 2005; Maunder et al., 2006; Renaud, 2008). This situation was also attributed to avoidant attached individuals’ tendency to suppress their stress symptoms, even though they actually, physically experience stress (Hunter et al., 2016; Teeruthroy & Bhowon, 2012). According to the current study’s results, the previously reported absence of a significant relationship between avoidant attachment and stress symptoms might be due to, not taking into consideration the possible moderating or mediating roles of some of the other critical variables, such as the ones used in this study.

The finding that anxious attachment is directly and indirectly, related to stress-related variables, including self-concept or self-perception, suggests that attachment is a key feature in describing stress. At the same time, it is assumed that the proposed model provides a useful and holistic view to understand how attachment relates to stress. Increased levels of anxious and avoidant attachment were found to be associated with increased levels of negative self-concept. It may be that individuals with a negative self-concept, tend to have a more helpless and submissive attitude, less self-confidence, and less optimistic attitudes towards the stressful events they experience. Lazarus and Folkman (1984) stated that, when individuals encounter a stressful situation, they make secondary appraisals about whether their resources are sufficient or not to cope with this stressful event, and that the resulting calculation/conclusion determines their next behavior. Going along with this argument, it can be stated that, the appraisals of the individual regarding the sufficiency of his/her resources, might be mediated by their self-perception and attachment style.

Our results showed that the anxiously attached individuals, regardless of their perceptions of themselves, use less effective and more ineffective coping strategies. Moreover, anxious attachment styles have a greater statistical power on the use of ineffective coping strategies. In other words, the level of anxious attachment effects coping with stressful situations in a negative way. These findings regarding attachment and coping strategies are consistent with the findings reported in previous studies (Hawkins et al., 2007; Li, 2008; Seiffge-Krenke & Beyers, 2005; Schottenbauer et al., 2006).

Another important finding of the current study is that, while effective coping was found to be significantly related to stress symptoms (not to stress factors), ineffective coping was found to be significantly and indirectly related to stress factors (not stress symptoms). It can be argued that if an individual thinks that, his/her resources are sufficient for effective coping, the perceived stress symptoms will be lower. On the other hand, it can also be asserted that considering oneself as incompetent and helpless to cope with stress, would lead one to evaluate the encountered events as more stressful, which in turn causes the individual to experience more stress symptoms. Despite the fact that in the related literature, the use of effective coping strategies was associated with lower stress and the use of ineffective coping methods was significantly associated with higher stress (Austin et al., 2005; Lopez et al., 2001; Şahin et al., 2009), the current finding of the absence of a significant relationship between effective coping and perceived stress factors is a subject that needs to be investigated in more detail in future studies.

Our results show little gender differences in research variables. There was no gender effect on stress symptoms. Avoidant attachment and effective coping were only found poorly related to gender. Males seem to have a tendency to use a little bit more avoidant attachment style and effective coping ways. The finding of avoidant attachment and gender relationship is consistent with previous research (Del Giudice, 2011). This tendency might depend on females being more social than males. Effective coping and gender relationship also consistent with the literature. Our results show that males tend to use more problem focus coping style than females as Folkman and Lazarus (1980) indicated.

Considering that the modern individual's current stress is originated from social threats, rather than physical ones, it is highly probable to argue that there is a decisive effect of the attachment styles on the perceived stress. Sapolsky (2004) suggests that the lack of social relations, associated with a lower lifetime expectancy and more diseases; and that stress caused by social isolation, negatively effects health by suppressing the immune system. In previous studies, it has been reported that both anxious and avoidant attachment styles are positively related depression, anxiety, and anger (Wei et al., 2003). Insecurely attached individuals experience higher levels of various psychological and physiological symptoms compared to securely attached ones (Kemp & Neimeyer, 1999; Muris et al., 2001; Pielage et al., 2000; Priel & Shamai, 1995; Schottenbauer et al., 2006; Watt et al., 2005). Based on the current research findings; it can be argued that individuals with anxious attachment, perceive situations as more stressful, and instead of more effective coping strategies, they tend to use helpless and submissive strategies and consequently report more stress symptoms.

In summary, it can be asserted that insecure attachment styles (especially anxious attachment), self-perception and strategies used in coping with stress, have meaningful predictor power in explaining perceived stress factors and stress symptoms. Individual’s attachment style is an important factor in understanding the concept of stress since it can predict self-concept, coping methods, perceived stress factors and ultimately experienced stress symptoms. The main contribution of this study to the literature is that the indirect effect of anxious attachment on stress through self-concept and coping is more than its direct effect on stress. Anxious attachment style negatively effects self-concept and impair coping abilities which weaken the individual in the face of stressful events and leads to perceive more stress factors and experience more stress.

While interpreting the findings of the current study, the limitations should also be considered. It should be noted that a common method variance and social desirability bias might exist since the research data was collected by means of a cross-sectional method and through self-report measures. Also, there may be a limitation to generalize the findings since the participants were all university students. However, it is assumed that since the participants were a homogeneous group regarding age and education, the effects of some possible environmental variables, not covered in the study could have been controlled. Future research is needed for different age groups or occupations reveal more generalizable results.

## References

[r1] Aaronson C. J. Bender D. S. Skodol A. E. Gunderson J. G. (2006). Comparison of attachment styles in borderline personality disorder and obsessive-compulsive personality disorder. The Psychiatric Quarterly, 77, 69–80. doi:. 10.1007/s11126-006-7962-x 16397756

[r2] Ainsworth, M. D. S., Blehar, M. C., Waters, E., & Wall, S. (1978). *Patterns of attachment: A study of the strange situations*. Hillsdale, NJ, USA: Lawrence Erlbaum.

[r3] Arbuckle, J. L. (2014). *Amos 23.0 User's Guide*. Chicago, IL, USA: IBM SPSS.

[r4] Austin V. Shah S. Muncer S. (2005). Teacher stress and coping strategies used to reduce stress. Occupational Therapy International, 12, 63–80. doi:. 10.1002/oti.16 16136865

[r5] Avşaroğlu S. Üre Ö. (2007). Üniversite öğrencilerinin karar vermede özsaygı, karar verme ve stresle başa çıkma stillerinin benlik saygısı ve bazı değişkenler açısından incelenmesi [Examination of self-esteem, decision making and stress coping styles of university students in terms of self-esteem and some variables] Selçuk University Social Sciences Institute journal, 18, 85-100.

[r6] Baron R. M. Kenny D. A. (1986). The moderator-mediator variable distinction in social psychological research: Conceptual, strategic and statistical considerations. Journal of Personality and Social Psychology, 51, 1173–1182. doi:. 10.1037/0022-3514.51.6.1173 3806354

[r7] Bartholomew K. Horowitz L. M. (1991). Attachment styles among young adults: A test of a four-category model. Journal of Personality and Social Psychology, 61, 226–244. 10.1037/0022-3514.61.2.226 1920064

[r8] Basut E. Erden E. G. (2005). Evaluation of stress symptoms and coping patterns of adolescents with and without criminal attempts. Turkish Journal of Child and Adolescent Mental Health, 12, 48–55.

[r9] Bowlby, J. (1973). *Attachment and Loss: Vol. 2. Separation: Anxiety and anger.* New York, NY, USA: Basic Books.

[r10] Brennan, K. A., Clark, C. L., & Shaver, P. R. (1998). Self-report of measurement of adult attachment: An integrative overview. In J. A. Simpsons & W. S. Rholes (Eds.), *Attachment theory and close relationship* (pp. 46-76). New York, NY, USA: Guilford.

[r11] Burmaoğlu S. Polat M. Meydan C. H. (2013). Relational analysis methods in organizational behavior literature and an investigation on the use of mediational models in Turkish literature. Anadolu University Journal of Social Sciences, 13, 13–26.

[r12] Büyüköztürk, Ş. (2010). *Sosyal bilimler için veri analizi el kitabı* [Data analysis handbook for social sciences] (12th ed.). Ankara, Turkey: Pegem Akademi.

[r13] Carnelley K. B. Pietromonacó P. R. Jaffe K. (1994). Depression, working models of others, and relationship functioning. Journal of Personality and Social Psychology, 66, 127–140. doi:. 10.1037/0022-3514.66.1.127 8126643

[r14] Damarlı, Ö. (2006). *The relationship between attachment styles, gender roles, and self-concept in adolescents* (Unpublished master’s thesis). Ankara University Social Sciences Institute, Ankara, Turkey.

[r15] Del Giudice M. (2011). Sex differences in romantic attachment: A meta-analysis. Personality and Social Psychology Bulletin, 37(2), 193–214. doi:. 10.1177/0146167210392789 21239594

[r16] Derogatis, L. R. (1992). *The Brief Symptoms Inventory (BSI): Administration, scoring, and procedures manual II*. Baltimore, MD, USA: Clinical Psychometric Research.

[r17] Doron G. Kyrios M. (2005). Obsessive-compulsive disorder: A review of possible specific internal representations within a broader cognitive theory. Clinical Psychology Review, 25, 415–432. doi:. 10.1016/j.cpr.2005.02.002 15885864

[r18] Elwood L. S. Williams N. L. (2007). PTSD-Related cognitions and romantic attachment style as moderators of psychological symptoms in victims of interpersonal trauma. Journal of Social and Clinical Psychology, 26, 1189–1209. doi:. 10.1521/jscp.2007.26.10.1189

[r19] Folkman S. Lazarus R. (1980). An Analysis of coping in a middle-aged community sample. Journal of Health and Social Behavior, 21, 219–239. 10.2307/2136617 7410799

[r20] Folkman S. Lazarus R. S. Gruen R. J. Delongis A. (1986). Appraisal, coping, health status, and psychological symptoms. Journal of Personality and Social Psychology, 50, 571–579. doi:. 10.1037/0022-3514.50.3.571 3701593

[r21] Fortuna K. Roisman G. I. (2008). Insecurity, stress, and symptoms of psychopathology: Contrasting results from self-reports versus interviews of adult attachment. Attachment & Human Development, 10, 11–28. doi:. 10.1080/14616730701868571 18351491

[r22] Fraley R. C. Shaver P. R. (2000). Adult romantic attachment: Theoretical developments, emerging controversies, and unanswered questions. Review of General Psychology, 4, 132–154. doi:. 10.1037/1089-2680.4.2.132

[r23] Gilbert P. Allan S. Trent D. (1991). A social comparison scale: Psychometric properties and relationship to psychopathology. Personality and Individual Differences, 19, 293–299. . 10.1016/0191-8869(95)00086-L

[r24] Greene J. W. Walker L. S. Hickson G. Thompson J. (1985). Stressful life events and somatic complaints in adolescents. Pediatrics, 75, 19–22. 3966041

[r25] Gürbüz, S., & Şahin, F. (2016). *Sosyal Bilimlerde Araştırma Yöntemleri Felsefe-Yöntem-Analiz* [Research Methods in Social Sciences Philosophy-Method-Analysis] (3rd ed.). Ankara, Turkey: Seçkin.

[r26] Hambleton, R. K., Swaminathan, H., & Rogers, H. J. (1991). *Fundamentals of item response theory*. Thousand Oaks, CA, USA: SAGE.

[r27] Hankin B. L. Kassel J. D. Abela J. R. Z. (2005). Adult attachment dimensions and specificity of emotional distress symptoms: Prospective investigations of cognitive risk and interpersonal stress generation as a mediating mechanism. Personality and Social Psychology Bulletin, 31, 136–151. doi:. 10.1177/0146167204271324 15574668

[r28] Hawkins A. C. Howard R. A. Oyebode J. Y. (2007). Stress and coping in hospice nursing staff: The impact of attachment styles. Psycho-Oncology, 16, 563–572. doi:. 10.1002/pon.1064 17004295

[r29] Hayes F. (2009). Beyond Baron and Kenny: Statistical mediation analysis in the new millennium. Communication Monographs, 76, 408–420. doi:. 10.1080/03637750903310360

[r30] Hunter, J., Maunder, R., & Le, T. L. (2016). Fundamentals of attachment theory. In J. Hunter & R. Maunder (Eds.), *Improving patient treatment with attachment theory: A guide for primary care practitioners and specialists* (pp. 9-26). Cham, Switzerland: Springer.

[r31] Jellema A. (2000). Insecure attachment states: Their relationship to borderline and narcissistic personality disorders and treatment process in cognitive analytic therapy. Clinical Psychology & Psychotherapy, 7, 138–154. doi:. 10.1002/(SICI)1099-0879(200005)7:2<138::AID-CPP231>3.0.CO;2-9

[r32] Kemp M. A. Neimeyer G. J. (1999). Interpersonal attachment: Experiencing, expressing, and coping with stress. Journal of Counseling Psychology, 46, 388–394. doi:. 10.1037/0022-0167.46.3.388

[r33] Kulaç, Ö. (2004). *The comparison of native and international cadets in the life of war academy, according to stress factors* (Unpublished master’s thesis). KHO Defence Sciences Institute, Ankara, Turkey.

[r34] Lazarus R. S. (1993). From psychological stress to the emotions: A history of changing outlooks. Annual Review of Psychology, 44, 1–22. doi:. 10.1146/annurev.ps.44.020193.000245 8434890

[r35] Lazarus, R. S., & Folkman, S. (1984). *Stress, appraisal, and coping*. New York, NY, USA: Springer.

[r36] Lazarus, R. S., & Folkman, S. (1986). Cognitive theories of stress and the issue of circularity. In M. H. Appley & R. Trumbull (Eds.), *Dynamics of stress. Physiological, psychological, and social perspectives* (pp. 63-80). Boston, MA, USA: Springer.

[r37] Lee M. Larson R. (1996). Effectiveness of coping in adolescence: The case of Korean examination stress. International Journal of Behavioral Development, 19, 851–869. doi:. 10.1177/016502549601900410

[r38] Li M.-H. (2008). Relationship among stress coping, secure attachment, and the trait of resilience among Taiwanese college students. College Student Journal, 42, 312–325.

[r39] Lopez F. G. Mauricio A. M. Gormley B. Simko T. Berger E. (2001). Adult attachment orientation and college student distress: The mediating role of problem coping styles. Journal of Counseling and Development, 79, 459–464. doi:. 10.1002/j.1556-6676.2001.tb01993.x

[r40] McCarthy C. J. Lambert R. Moller N. P. (2006). Preventive resources and emotion regulation expectancies as mediators between attachment and college students’ stress outcomes. International Journal of Stress Management, 13, 1–22. doi:. 10.1037/1072-5245.13.1.1

[r41] Maunder R. G. Lancee W. J. Nolan R. P. Hunter J. J. Tannenbaum D. W. (2006). The relationship of attachment insecurity to subjective autonomic function during standardized acute stress in healthy adults. Journal of Psychosomatic Research, 60, 283–290. doi:. 10.1016/j.jpsychores.2005.08.013 16516661

[r42] Mauricio A. M. Tein J. Y. Lopez F. G. (2007). Borderline and antisocial personality scores as mediators between attachment and intimate partner violence. Violence and Victims, 22, 139–157. doi:. 10.1891/088667007780477339 17479552

[r43] Merlo L. J. Lakey B. (2007). Trait and social influences in the links among adolescent attachment, depressive symptoms, and coping. Journal of Clinical Child and Adolescent Psychology, 36, 195–206. doi:. 10.1080/15374410701277846 17484692

[r44] Meydan, C. H., & Şeşen, H. (2011). *Yapısal eşitlik modellemesi AMOS uygulamaları* [Structural equation modeling AMOS applications]. Ankara, Turkey: Detay Publishing.

[r45] Morris, D. (1982). Attachment and intimacy. In M. Fisher & G. Stricker (Eds.), *Intimacy* (pp. 305-323). New York, NY, USA: Plenum.

[r46] Muris P. Meesters C. Melick M. V. Zwambag L. (2001). Self-reported attachment style, attachment quality, and symptoms of anxiety and depression in young adolescents. Personality and Individual Differences, 30, 809–818. doi:. 10.1016/S0191-8869(00)00074-X

[r47] Neria Y. Steinmetz S. G. Koenen K. Levinovsky L. Zakin G. Dekel R. (2001). Do attachment and hardiness relate to each other and to mental health in real life stress? Journal of Social and Personal Relationships, 18, 844–858. doi:. 10.1177/0265407501186006

[r48] Park C. L. Folkman S. (1997). Meaning in the context of stress and coping. Review of General Psychology, 1, 115–144. doi:. 10.1037/1089-2680.1.2.115

[r49] Pielage S. Gerlsma C. Schaap C. (2000). Insecure attachment as a risk factor for psychopathology: The role of stressful events. Clinical Psychology & Psychotherapy, 7, 296–302. doi:. 10.1002/1099-0879(200010)7:4<296::AID-CPP262>3.0.CO;2-8

[r50] Preacher K. J. Hayes A. F. (2008). Asymptotic and resampling strategies for assessing and comparing indirect effects in multiple mediator models. Behavior Research Methods, 40, 879–891. doi:. 10.3758/BRM.40.3.879 18697684

[r51] Priel B. Shamai D. (1995). Attachment style and perceived social support: Effects on affect regulation. Personality and Individual Differences, 19, 235–241. doi:. 10.1016/0191-8869(95)91936-T

[r52] Renaud E. F. (2008). The attachment characteristics of combat veterans with PTSD. Traumatology, 14, 1–12. doi:. 10.1177/1534765608319085

[r53] Şahin, N. H., Akgiray, A., Alkan, E., Arı, Ö., Bayezit, S., Kent, S., … Üstün, B. (2005, April). *Üniversite öğrencileri stres faktörleri: Özgün bir ölçek* [Stress factors among university students: An original scale]. Presented at the II. Prof. Dr. Işık Savaşır Clinical Psychology Symposium, Ankara, Turkey, METU.

[r54] Şahin N. H. Basım H. N. Akkoyun N. (2011). A-Tipi Kişilik ve Stres İlişkisinde Üç Önemli Bileşen: Öfke, Etkisiz Başa Çıkma ve İş Saplantısı [Three critical components in the Type-A and stress relationship: Anger, ineffective coping, and obsession with work] Turkish Journal of Psychology, 26, 31–44.

[r55] Şahin N. H. Durak A. (1994). Kısa Semptom Envanteri (Brief Symptom Inventory-BSI): Türk Gençleri [A study of the brief symptom inventory in Turkish youth] Turkish Journal of Psychology, 9, 44–56. 12794665

[r56] Şahin N. H. Durak A. (1995). Stresle Basaçıkma Tarzları Ölçegi: Üniversite Ögrencileri Için Uyarlanması [A brief coping styles inventory for university students] Turkish Journal of Psychology, 10, 56–73.

[r57] Şahin, N. H., Durak, A., & Şahin, N. (1993). *Bir grup banka personelinde iş doyumu ve stres* [Job satisfaction and stress in a group of bank staff]. Unpublished research report.

[r58] Şahin N. H. Güler M. Basım H. N. (2009). The relationship between cognitive intelligence, emotional intelligence, coping and stress symptoms in the context of Type A personality pattern. Turkish Journal of Psychiatry, 20, 243–254. 19757224

[r59] Şahin, N. H., & Şahin, N. (1992, June). *Guilt, shame, and depression in adolescence.* Paper presented at the World Congress of Cognitive Therapy, Toronto, Canada.

[r60] Sapolsky, R. (2004). *Why zebras don't get ulcers: An updated guide to stress, stress-related disease and coping* (3rd ed.). New York, NY, USA: Henry Holt and Company.

[r61] Savaşır, I., & Şahin, N. H. (1997). *Bilişsel davranışçı terapilerde değerlendirme: sık kullanılan ölçekler* [Assessment in cognitive behavioral therapies: commonly used scales]. Ankara, Turkey: Turkish Psychological Association.

[r62] Schottenbauer M. A. Klimes-Dougan B. Rodriguez B. F. Arnkoff D. B. Glass C. R. LaSalle H. (2006). Attachment and affective resolution following a stressful event: General and religious coping as possible mediators. Mental Health, Religion & Culture, 9, 448–471. doi:. 10.1080/13694670500440684

[r63] Seiffge-Krenke I. S. Beyers W. (2005). Coping trajectories from adolescence to young adulthood: Links to attachment state of mind. Journal of Research on Adolescence, 15, 561–582. doi:. 10.1111/j.1532-7795.2005.00111.x

[r64] Selçuk E. Günaydin G. Sümer N. Uysal A. (2005). Yetişkin Bağlanma Boyutları İçin Yeni Bir Ölçüm: Yakın İlişkilerde Yaşantılar Envanteri-II’nin Türk Örnekleminde Psikometrik Açıdan Değerlendirilmesi [A new scale developed to measure adult attachment dimensions: Experiences in Close Relationships-Revised (ECR-R) - Psychometric evaluation in a Turkish sample] Turkish Psychological Articles, 8, 1–11.

[r65] Shrout P. E. Bolger N. (2002). Mediation in experimental and nonexperimental studies: New procedures and recommendations. Psychological Methods, 7, 422–445. doi:. 10.1037/1082-989X.7.4.422 12530702

[r66] Sığrı Ü. (2007). Geçici ve Daimi Personelin Stres Faktörlerinin, Belirtilerinin, Yatkınlıklarının ve Stresle Baş Etme Tarzlarının Mukayeseli Analizi [Comparative analysis of stress factors, symptoms, tendencies, and stress coping styles of the conscripted and professional personnel] Journal of ONERI, 7, 177–188.

[r67] Sobel, M. E. (1982). Asymptotic confidence intervals for indirect effects in structural equations models. In S. Leinhart (Ed.), *Sociological methodology* (pp. 290-312). San Francisco, CA, USA: Jossey-Bass.

[r68] Sperling, M. B., & Berman, W. H. (1994). *Attachment in adults*. New York, NY, USA: The Guilford Press.

[r69] Sümer N. (2006). Yetişkin bağlanma ölçeklerinin kategoriler ve boyutlar düzeyinde karşılaştırılması [Categorical and dimensional comparison of the adult attachment measures] Turkish Journal of Psychology, 21, 1–22.

[r70] Sümer N. Güngör D. (1999). The impact of perceived parenting styles on attachment styles, self-evaluations, and close relationships. Turkish Journal of Psychology, 14, 35–62.

[r71] Teeruthroy V. T. Bhowon U. (2012). Romantic relationships among young adults: An attachment perspective. International Journal of Humanities and Social Science, 2, 145–155.

[r72] Tuğrul C. (1994). Alkoliklerin çocuklarının aile ortamlarındaki stres kaynakları, etkileri ve stresle başa çıkma yolları [Alcoholics’ children's stress sources in family settings, their effects and ways of coping with stress] Turkish Journal of Psychology, 9, 57–73.

[r73] Tully A. (2004). Stress, sources of stress and ways of coping among psychiatric nursing student. Journal of Psychiatric and Mental Health Nursing, 11, 43–47. doi:. 10.1111/j.1365-2850.2004.00682.x 14723638

[r74] Watt M. C. McWilliams L. A. Campbell A. G. (2005). Relation between anxiety sensitivity and attachment style dimensions. Journal of Psychopathology and Behavioral Assessment, 27, 191–200. doi:. 10.1007/s10862-005-0635-5

[r75] Wei M. Heppner P. P. Mallinckrodt B. (2003). Perceived coping as a mediator between attachment and psychological distress: A structural equation modeling approach. Journal of Counseling Psychology, 50, 438–447. doi:. 10.1037/0022-0167.50.4.438

[r76] Wei M. Mallinckrodt B. Larson L. M. Zakalik R. A. (2005). Adult attachment, depressive symptoms, and validation from self versus others. Journal of Counseling Psychology, 52, 368–377. doi:. 10.1037/0022-0167.52.3.368

